# The Extirpation of Mammary Carcingma

**Published:** 1890-07

**Authors:** 


### THE EXTIRPATION OF MAMMARY CARCINGMA.

Dr. Lewis S. Pilcher (*Brooklyn Medical Journal*, April, 1890), in discussing operations for the removal of mammary cancer, says that the researches of Heidenhain into the causes of local relapse of cancer after amputation of the breast seem to be of the greatest importance, and particularly his demonstration that in two-thirds of all cases of mammary cancer numerous cancerous deposits are to be found in the lymphatics which pass through the layers of retro-mammary adipose tissue to the pectoral fascia beneath, so that though the visible tumor may be freely movable over this fascia, nevertheless cancerous elements may be already present in the lymphatics of the fascia. These lymphatics accompany the blood vessels which penetrate the muscle and thus become the channels of infection. Security from recurrence cannot, therefore, be obtained even in those cases in which the mammary growth is still apparently limited to the gland, except by taking away with it both the underlying pectoral fascia and a thick layer of the muscular substance itself. The practical lesson, corroborated by all the experience of the past in the almost certain recurreace after extirpation, is that no operation for the removal of cancer of the breast can be considered radical and trustworthy which does not combine free ablation of the breast and its overlying integument, with entire cleaning out of the glands and connective tissue of the axilla, and removal of the pectoral fascia and superficial layer of the portion of the pectoral muscle lying underneath the gland. Dr. Pilcher is ready to accept also, as a working hypothesis for the present, the suggestion as to the complete extirpation of the whole pectoral muscle when any part of it has become involved.—*Med. News.*

---

OLD LADY—"I'd like to buy som plasters, young fellow."

Drug Clerk—"Yes, ma'am ; porous ?

Old Lady—"Do you s'pose I want to ketch my death of cold? Let's see yer winter styles.'—*Judge.*

---

During an attack of gout, the urine is deficient in uric acid and urates; *after* the attack is over, they are in excess.—*Med. World.*

